# Supplementary material for: REACT study protocol: resilience after the COVID-19 threat (REACT) in adolescents
Source: BMJ Open. 2021 Jan 20;11(1):e042824. doi: 10.1136/bmjopen-2020-042824 (PMC7818833; doi:10.1136/bmjopen-2020-042824)
Supplement: Author's manuscript [file bmjopen-2020-042824.draft_revisions.pdf]

BMJ Open

BMJ Open is committed to open peer review. As part of this commitment we make the peer review history of every article we publish publicly available.

When an article is published we post the peer reviewers' comments and the authors' responses online. We also post the versions of the paper that were used during peer review. These are the versions that the peer review comments apply to.

The versions of the paper that follow are the versions that were submitted during the peer review process. They are not the versions of record or the final published versions. They should not be cited or distributed as the published version of this manuscript.

BMJ Open is an open access journal and the full, final, typeset and author-corrected version of record of the manuscript is available on our site with no access controls, subscription charges or pay-per-view fees (http://bmjopen.bmj.com).

If you have any questions on BMJ Open's open peer review process please email info.bmjopen@bmj.com

BMJ Open

The Resilience after the COVID-19 Threat (REACT) study: a prospective study of young adult wellbeing in response to the pandemic.

Journal:	*BMJ Open*
Manuscript ID	bmjopen-2020-042824
Article Type:	Protocol
Date Submitted by the Author:	16-Jul-2020
Complete List of Authors:	Smith, Alicia; University of Cambridge, Department of Psychiatry; University of Cambridge, MRC Cognition and Brain Sciences Unit<br>Moreno-López, Laura; University of Cambridge School of Clinical Medicine, Department of Psychiatry<br>Davidson, Eugenia; University of Cambridge, Department of Psychiatry; University of Exeter, College of Medicine and Health<br>Dauvermann, Maria; University of Cambridge, Department of Psychiatry; Massachusetts Institute of Technology, McGovern Institute for Brain Research<br>Orellana, Sofia; University of Cambridge, Department of Psychiatry<br>Soneson, Emma ; University of Cambridge Department of Psychiatry, Department of Psychiatry<br>Ioannidis, Konstantinos; University of Cambridge School of Clinical Medicine, Department of Psychiatry; Cambridgeshire and Peterborough NHS Foundation Trust<br>Kaser, Muzaffer; University of Cambridge School of Clinical Medicine; Cambridgeshire and Peterborough NHS Foundation Trust<br>van Harmelen, Anne-Laura; University of Cambridge School of Clinical Medicine, Department of Psychiatry; Leiden University, Education and Child Studies
Keywords:	COVID-19, Neurobiology < NATURAL SCIENCE DISCIPLINES, PSYCHIATRY

SCHOLARONE™
Manuscripts

I, the Submitting Author has the right to grant and does grant on behalf of all authors of the Work (as defined in the below author licence), an exclusive licence and/or a non-exclusive licence for contributions from authors who are: i) UK Crown employees; ii) where BMJ has agreed a CC-BY licence shall apply, and/or iii) in accordance with the terms applicable for US Federal Government officers or employees acting as part of their official duties; on a worldwide, perpetual, irrevocable, royalty-free basis to BMJ Publishing Group Ltd ("BMJ") its licensees and where the relevant Journal is co-owned by BMJ to the co-owners of the Journal, to publish the Work in this journal and any other BMJ products and to exploit all rights, as set out in our licence.

The Submitting Author accepts and understands that any supply made under these terms is made by BMJ to the Submitting Author unless you are acting as an employee on behalf of your employer or a postgraduate student of an affiliated institution which is paying any applicable article publishing charge ("APC") for Open Access articles. Where the Submitting Author wishes to make the Work available on an Open Access basis (and intends to pay the relevant APC), the terms of reuse of such Open Access shall be governed by a Creative Commons licence – details of these licences and which Creative Commons licence will apply to this Work are set out in our licence referred to above.

Other than as permitted in any relevant BMJ Author's Self Archiving Policies, I confirm this Work has not been accepted for publication elsewhere, is not being considered for publication elsewhere and does not duplicate material already published. I confirm all authors consent to publication of this Work and authorise the granting of this licence.

For peer review only

The Resilience after the COVID-19 Threat (REACT) study: a prospective study of young adult wellbeing in response to the pandemic.

Alicia J. Smith[1,2*], Laura Moreno-López[3], Eugenia Davidson[4,5], Maria R. Dauvermann[6,7], Sofia Orellana[8], Emma Soneson[9], Konstantinos Ioannidis[10,11], Muzaffer Kaser[12,13] & Anne-Laura van Harmelen[14,15*]

*Corresponding authors

For peer review only

Word count = 3310
Abstract = 300

[1] Department of Psychiatry, University of Cambridge, Douglas House, 18b Trumpington Rd, Cambridge CB2 8AH, UK. as2804@medschl.cam.ac.uk
[2] MRC Cognition and Brain Sciences Unit, University of Cambridge, 15 Chaucer Road, Cambridge, CB2 7EF, UK. alicia.smith@mrc-cbu.cam.ac.uk
[3] Department of Psychiatry, University of Cambridge, Douglas House, 18b Trumpington Rd, Cambridge CB2 8AH, UK. lm618@medschl.cam.ac.uk
[4] Department of Psychiatry, University of Cambridge, Douglas House, 18b Trumpington Rd, Cambridge CB2 8AH, UK. ed555@medschl.cam.ac.uk
[5] College of Medicine and Health, University of Exeter, Stocker Rd, Exeter, EX4 4PY, UK. ed555@medschl.cam.ac.uk
[6] Department of Psychiatry, University of Cambridge, Douglas House, 18b Trumpington Rd, Cambridge CB2 8AH, UK. md874@medschl.cam.ac.uk
[7] McGovern Institute for Brain Research, Massachusetts Institute of Technology, Cambridge, USA. md874@medschl.cam.ac.uk
[8] Department of Psychiatry, University of Cambridge, Douglas House, 18b Trumpington Rd, Cambridge CB2 8AH, UK. sdco2@medschl.cam.ac.uk
[9] Department of Psychiatry, University of Cambridge, Douglas House, 18b Trumpington Rd, Cambridge CB2 8AH, UK. es703@medschl.cam.ac.uk
[10] Department of Psychiatry, University of Cambridge, Douglas House, 18b Trumpington Rd, Cambridge CB2 8AH, UK. konstantinos.ioannidis@cpft.nhs.uk
[11] Cambridgeshire and Peterborough NHS Foundation Trust, Fulbourn, UK. konstantinos.ioannidis@cpft.nhs.uk
[12] Department of Psychiatry, University of Cambridge, Douglas House, 18b Trumpington Rd, Cambridge CB2 8AH, UK. mk708@medschl.cam.ac.uk
[13] Cambridgeshire and Peterborough NHS Foundation Trust, Fulbourn, UK. mk708@medschl.cam.ac.uk
[14] Department of Psychiatry, University of Cambridge, Douglas House, 18b Trumpington Rd, Cambridge CB2 8AH, UK. Av391@medschl.cam.ac.uk
[14] Institute of Education and Child Studies, Leiden University, Pieter de la Court building, 4th floor Wassenaarseweg 52, 2333 AK Leiden. a.van.harmelen@fsw.leidenuniv.nl

ABSTRACT

Introduction: Coronavirus disease 2019 (COVID-19) and associated social isolation and stress, may have significant mental health effects for many individuals, including elevated symptoms of post-traumatic stress, anxiety and depression. These effects are thought to disproportionally affect vulnerable populations already at risk for psychopathology, such as youth with a history of childhood adversity (CA). Research is required to reveal which factors act to buffer the negative impact of COVID-19 related stress and isolation in these vulnerable youth. The Resilience after the COVID-19 Threat (REACT) study will assess whether emotion regulation capacity, inflammation and neuroimmune responses to acute stress induced in the laboratory prior to the pandemic predicts changes in behaviour and responses to social isolation and COVID-19 related stress in a sample of adolescents with CA. We aim to elucidate the mechanisms that enable vulnerable individuals to maintain or regain good mental health when confronted with COVID-19. **Methods and analysis:** We will follow-up with a sample of adolescents (aged 16-26) who have experienced CA and recently partaken in the Resilience after Individual Stress Exposure (RAISE) study for which we assessed emotion regulation, neural and immune stress responses to an established acute stress task. Questionnaires were completed at the start of lockdown ('baseline'; April 2020) and will be readministered at three (July 2020) and six months (October 2020) thereafter, providing crucial longitudinal measurements following the phases of the pandemic progression and government response. The questionnaires assess 1) mental health, 2) number and severity of life events, 3) physical health, 4) perception of stress, and 5) loneliness and friendship support. We will use structural equation modelling to examine whether individual differences at baseline are associated with changes in behaviour and responses to social isolation and COVID-19 related stress. **Ethics and dissemination:** Ethical approval has been granted by Cambridge Psychology Research Ethics Committee (PRE.2020.037).

For peer review only - http://bmjopen.bmj.com/site/about/guidelines.xhtml

Key words: Resilience; Stress; COVID-19; Childhood adversity; Maltreatment; Adolescence

ARTICLE SUMMARY

STRENGTHS AND LIMITATIONS

- Utilising an integrated bio-psycho-social design, we will be able to examine the relations and interrelations of emotion regulation, neural and immune responses in predicting health and wellbeing across the trajectory of the COVID-19 lockdown.
- Due to the prospective design, we will be able to examine intra-individual *changes* in wellbeing and stress perceptions before, during and after COVID-19.
- Due to deep neuro-immune phenotyping prior to COVID-19, we will be able to examine whether individual differences in neuro-immune factors predict wellbeing and stress perception in response to COVID-19.

For peer review only

For peer review only - http://bmjopen.bmj.com/site/about/guidelines.xhtml

1. INTRODUCTION

The recent outbreak of a severe acute respiratory syndrome caused by the Coronavirus disease 2019 (COVID-19) originating in China has escalated globally (1). Governments have imposed nation-wide measures of disease containment and urged their citizens to practice social distancing and isolation. As a consequence, millions of people worldwide are currently experiencing unprecedented periods of social isolation and stress (2). Consequences of the social isolation and COVID-related stress will likely result in increased rates of depression, acute and post-traumatic stress and clinical anxiety, especially in already vulnerable populations (3, 4) and in demographically mediated ways (5).

Negative impact of the current pandemic is thought to disproportionally affect populations already at risk for psychopathology, such as youth with a history of childhood adversity (CA) (3, 6). CA, comprising emotional, sexual and/or physical abuse, emotional and/or physical neglect, marital distress/conflict, parental mental health problems and/or parental alcohol dependence, violence and/or aggressive behaviour prior to the age of 16, is one of the strongest predictors of poor mental health and wellbeing in adulthood (7-9).

Despite evidence of the detrimental effects of CA on mental health, a significant proportion of individuals show little or no long-term negative sequelae; they display 'resilience' (10). Currently, our understanding of *resilience* has been mostly limited to studying outcome after individual-level trauma or commonly experienced catastrophes such as terror attacks or natural disasters. So far, studies examining resilience have either focussed on [1] a trait like *capacity* that precedes adversity, [2] a dynamic *process* that unfolds during and after adversity, or [3] an *outcome* following adversity. Our recent resilience framework (10) combines these viewpoints and describes that *resilience* is the dynamic process of positive adaptation to stress. Whereas, resilient functioning refers to a positive outcome in the

For peer review only - http://bmjopen.bmj.com/site/about/guidelines.xhtml

aftermath of a stressor, and can be measured as better mental health and wellbeing than others with similar adverse experiences (11). Resilience and functioning outcomes are facilitated by *resilience factors*, protective factors residing on social (e.g., friendships), psychological (e.g., emotion regulation capacity), as well as neurobiological levels (e.g., downregulation of stress responses) that together underlie an individuals' capacity to adapt and recover from the impact of negative events and facilitate resilient functioning after CA (11-13). However, it is unknown how these factors interact to facilitate resilience to the COVID pandemic.

The psychological impact of the current pandemic on the general public and modifiable resilience factors that may explain the individual differences in young people's responses have been identified. In China, a cross-sectional study of young people aged 14-35 found that 40.4% of the sample reported having psychological problems and 14.4% presented post-traumatic stress disorder (PTSD) symptoms two weeks after the outbreak of COVID-19 (14). Recent work by Li et al. (15) on 4607 Chinese citizens aged 16 years and over suggested that individuals' cognitive appraisals and perceived severity of the COVID-19 threat was predictive of negative emotional and behavioural responses. Similarly, data from Europe has revealed a positive association between resilience to stress exposure during the COVID-19 lockdown and positive appraisal style in a sample of N=5000 adults (16). In a large UK sample, personality traits of compassion, conscientiousness, perfectionism, and extravertedness were associated with experiencing a more positive global (psycho-socio-economic) impact of the COVID-19 pandemic (5). In sum, emotion regulation is thought to be a critical resilience factor (17).

It has been reported that adversity experienced in childhood has potential detrimental effects on emotion regulation capacity and stress regulation through internal biological processes, such as altered inflammatory processes (i.e. changes in circulating lymphocyte count and proinflammatory markers) and neurobiological mechanisms (i.e. increased central

For peer review only - http://bmjopen.bmj.com/site/about/guidelines.xhtml

executive response and lower salience network activity) (12, 18-21). During exposure to stress, the body releases pro-inflammatory markers including high sensitivity C-reactive protein (hs-CRP) and cytokines, including interleukin (IL)-1β, IL-6 and tumor necrosis factor (TNF)-α (22). These cytokines play a key role in acute stress reactivity by activating the hypothalamic-pituitary-adrenal (HPA) axis to release glucocorticoids such as cortisol (23). In turn, cortisol suppresses the release of cytokines, playing an important anti-inflammatory role in acute stress recovery. While the priming on the immune system following acute stress likely represents an adaptive response, research suggests that acute stress causes an upregulation of inflammatory mediators in circulation and in the amygdala and a decrease in inflammatory mediators in the medial prefrontal cortex (mPFC) (24) – regions involved in executive functioning and emotion regulation (25, 26). The elevated release of proinflammatory markers in response to a chronic stressor, such as CA, has been associated with both structural and functional alterations in these brain regions (27). Furthermore, reduced volumes of the amygdala and the mPFC, as well as increased/decreased activation of these regions during executive and affective control tasks may in turn confer vulnerability to mood disorders such as depression and/or anxiety (28-30).

Research is required to understand how interrelated resilience factors, ranging from 'bottom-up' polygenetic influences to 'top-down' social environmental factors, can facilitate resilient functioning to new stress, such as COVID-19, after CA. The HPA axis, controlling stress reactions and immune functioning, is one system that inextricably intertwines with brain structure and function and may be linked to vulnerable or resilient responses to COVID-19. In the context of CA, cortisol responses to awakening and acute stress response differ for patients with and without psychopathology (31). Results of a recent meta-analysis revealed that in a healthy population, CA was associated with increased cortisol awakening response and lower baseline cortisol response (32). In the ongoing Resilience after Individual

For peer review only - http://bmjopen.bmj.com/site/about/guidelines.xhtml

Stress Exposure (RAISE) study we investigated how the HPA axis and immune system interrelate with brain structure and function, and social environment to facilitate resilient functioning by measuring emotion regulation capacity and stress responses to a well-validated functional magnetic resonance imaging (fMRI) task, the Montreal imaging stress task (MIST; (33)), in individuals with CA. In the current study, we have the unique opportunity to leverage the RAISE data to investigate how individual differences in emotion regulation and stress responses to previous laboratory-based stressors predicts individual differences in response to COVID-19 stress and isolation. By assessing individuals' responses before, during and after a universally experienced stressor, the REACT study will enable us to integrate and examine social, psychological and biological resilience factors across the trajectory and to predict resilience during stress and/or resilient functioning outcomes.

OBJECTIVES AND HYPOTHESIS

The aim of this study is to examine whether and how social support, emotion regulation capacity, baseline inflammation, and/or neuroimmune responses to laboratory-induced acute stress in adolescents predicts: 1) perceived stress, 2) emotional processing, 3) social behaviour, 4) general mental health, and 5) physical health across the trajectory of the COVID-19 restrictions and restructuring.

We hypothesise that social support, resilient emotion regulation and/or stress responses to previous laboratory-based stressors (both in the brain (i.e. increased central executive response and lower salience network activity) and in the periphery (i.e. lower levels of cortisol, IL-1b, IL-6, TNF-α and hs-CRP)) will be associated with greater resilient functioning as well as better mental and physical health and wellbeing (lower perceived

For peer review only - http://bmjopen.bmj.com/site/about/guidelines.xhtml

stress, lower loneliness and increased perception of peer support) across the trajectory of the COVID-19 pandemic (11, 12, 30, 34-37).

2. METHODS AND ANALYSIS

3.1. Study design

All participants have provided written informed consent to complete a set of eleven questionnaires at three time points: at baseline (April 2020), 3 months (July 2020) and 6 months (October 2020) thereafter. By distributing baseline questionnaires in April, a few weeks after the initial lockdown restrictions in the UK were imposed, the social restrictions serve as a timeline for us to measure the wider impact of the pandemic on society. Questionnaires sent at three and six months thereafter will measure responses as these restrictions are gradually eased. Beyond government measures, the COVID-19 pandemic has a pervasive impact on people's lives due to a substantial and reactive restructuring of society on multiple levels of ecology. The follow-up questionnaires will provide crucial longitudinal measurements, in the context of the impending socio-ecological change, following the phases of the pandemic progression and government response.

Patient and Public Involvement (PPI) was conducted prior to the RAISE study and feedback from the panel was utilised to improve the quality, relevance and impact of the REACT study.

3.2. Participants and recruitment

Participants were recruited from the RAISE study; an ongoing study carried out in the Department of Psychiatry at the University of Cambridge, which aims to study resilient functioning in adolescents who have experienced CA. In the RAISE study, participants complete an online assessment to assess psychological functioning, emotion regulation

For peer review only

capacity and early life experiences as well as an in-unit assessment at Addenbrooke's Hospital to assess neural and immune responses to laboratory-induced acute stress.

Inclusion criteria for the RAISE study were: aged 16-26 years inclusive, able and willing to give informed consent, able to speak, write, and understand English, body mass index (BMI) between 18.5 and 29.9 kg/m2, experienced CA (emotional, sexual and/or physical abuse, emotional and/or physical neglect, marital distress/conflict, parental mental health problems and/or parental alcohol dependence, violence and/or aggressive behaviour before the age of 16). Exclusion criteria for the RAISE study were: alcohol or substance use disorder within the past six months, current disorders likely to compromise the interpretation of the data (e.g. immunological disorders, cardiovascular disorders, endocrine and autoimmune disorders, malignancies or infections, or any other condition to be determined by the principal investigator or delegate), current medication likely to compromise the interpretation of immunological data (including, but not limited to, corticosteroids or any other substance to be determined by the principal investigator or delegate).

Participants for the RAISE study were recruited in Cambridge, UK, through advertisements in the general community (e.g., posters in hospitals, colleges, coffee shops) and on social media platforms (e.g., Facebook and Twitter advertisements). We also recruited from an existing database of participants who had previously taken part in the NeuroScience in Psychiatry Network (NSPN; N=2389) and had consented to being contacted for future studies.

3.3. Study procedure

All RAISE study participants who consented to being re-contacted for future studies were approached via email and provided with the participant information sheet. All interested

For peer review only - http://bmjopen.bmj.com/site/about/guidelines.xhtml

participants were asked to complete online assessments at three timepoints and were encouraged to contact the study team with any questions that they had.

Participants who expressed interest in taking part received an email with the secure links and instructions to complete the first online consent form and set of questionnaires. The links to the baseline questionnaires were sent at the beginning of April. The same questionnaires and computer tasks will be sent three and six months thereafter to follow-up any changes experienced by the participant.

All three assessments (baseline (April 2020), three month (July 2020) and six month follow-ups (October 2020)) will include the following self-report questionnaires:

COVID-19 Adolescent Symptom & Psychological Experience Questionnaire (38). Emotional, cognitive and social experiences related to COVID-19 will be addressed using the COVID-19 Adolescent Symptom & Psychological Experience Questionnaire (CASPE). The CASPE forms part of the research tracker and facilitator for Assessment of COVID-19 Experiences (ACE) for Adolescents developed by scientists at the University of Oregon (https://doi.org/10.17605/OSF.IO/PY7VG). The CASPE has a total of 38 items distributed in four major categories: 1) experience related to COVID-19 and symptoms, 2) emotional experience, 3) cognitive experience, and 4) social experience.

Life-Events Questionnaire (39). Number and severity of negative events experienced will be assessed with the Life Events Questionnaire (LEQ). Participants will rate 13 major life events which may have occurred during the preceding 18-month period including changes in school, illness, moving house, deaths and friendship difficulties. If affected by the event, respondents are asked to detail what happened and rate it on a scale of 1='very pleasant/happy' to 5='very unpleasant/sad/painful'. A higher score indicates a greater number of adverse life events experienced in the 18 months prior to the assessment.

For peer review only - http://bmjopen.bmj.com/site/about/guidelines.xhtml

Physical Health Questionnaire (PHQ) (40). Physical health will be assessed using a 14-item questionnaire that covers four areas of general physical health and areas that are often affected by stress (both acute and chronic), such as gastrointestinal problems, headaches, sleep disturbances, and respiratory illness. Higher scores indicates greater somatic health.

The Mood and Feelings Questionnaire (41). The Mood and Feelings Questionnaire (MFQ) is a 33-item instrument that was developed to assess mental health and wellbeing from two weeks prior and up to the date of the assessment. The MFQ has shown prognostic validity in both clinical and non-clinical samples (42, 43). Higher sum scores indicate higher levels of depressive symptoms.

The Revised Children's Manifest Anxiety Scale (44). The Revised Children's Manifest Anxiety Scale (RCMAS) is a 37-item instrument that assesses physiological anxiety, worry/oversensitivity, social concerns/concentration, and total anxiety score. A high score indicates higher levels of anxiety.

Warwick-Edinburgh Mental Wellbeing Scale (45). The Warwick-Edinburgh Mental Wellbeing Scale (WEMWBS) assesses responses to 14 positively worded statements to quantify mental wellbeing in the general population. Each item is graded by the respondent from 'none of the time' to 'all of the time'. Higher scores indicate greater wellbeing.

Strengths and Difficulties Questionnaire YR1 (46). The Strengths and Difficulties Questionnaire (SDQ) is a 25-item self-report measure, which is divided into five subscales. Four of the subscales measure difficulties and one subscale measures strengths, a prosocial behaviour. The difficulties subscales assess emotional symptoms (internalising symptoms), conduct problems, hyperactivity/inattention and peer relationship problems. The measure has been shown to have good psychometric properties in the age group that will be recruited for the current study (Cronbach's α of 0.80), as well as good sensitivity, specificity and

For peer review only - http://bmjopen.bmj.com/site/about/guidelines.xhtml

prospective utility. The total difficulties score is calculated by summing the scores of the four difficulties subscales. A high score indicates higher risk of clinically significant problems and can be used to identify likely cases of mental health problems. Conversely, a low score on the prosocial behaviour subscale indicates higher risk of clinically significant problems.

Perceived Stress Scale (47). The Perceived Stress Scale (PSS) is the most widely used psychological instrument for measuring the perception of stress. It is a 10-item measure used to assess the degree to which situations in one's life are appraised as stressful. Items are designed to assess how unpredictable, uncontrollable, and overloaded respondents find their lives. The scale also includes several direct queries about current levels of experienced stress. A higher sum score indicates higher perceived stress.

Revised UCLA Loneliness Scale (48). The Revised UCLA Loneliness Scale (R-UCLA) is a 20-item scale designed to measure subjective feelings of loneliness and social isolation. Participants rate each item on a scale from 1 (Never) to 4 (Often). Higher scores indicate a greater degree of loneliness.

Cambridge friendship questionnaire. The Cambridge friendship questionnaire (CFQ) is an 8-item questionnaire that assesses the quality and number of friendships. The CFQ has good measurement invariance and external validity, and has demonstrated ecological validity across two samples (49, 50). Higher scores indicate better perceived overall quality of friendships.

3.4. Statistical analysis plan

Responses during and after COVID-19 will be assessed using growth modelling analyses, a statistical technique implemented within a Structural Equation Modelling (SEM) framework, which allows for the estimation of subject-specific trajectories of change across time for a given set of variables of interest (51, 52). Consequently, implementing growth modelling

For peer review only - http://bmjopen.bmj.com/site/about/guidelines.xhtml

analyses, individual intercepts (baseline) and slopes (change) of trajectories for mental and physical health and wellbeing, perceived stress, loneliness and friendship support will be estimated. We will then input these parameter in regression analyses in order to determine whether they are associated with individual differences in emotion regulation capacity and/or neural and immune responses to stress in the laboratory. Across all models, we will add differences in social distancing behaviours and negative life events experienced as covariates, in addition to age, sex and BMI.

3. ETHICS AND RISKS

The study has been approved by the Cambridge Psychology Research Ethics Committee (PRE.2020.037). Ethical guidelines will be adhered to throughout the study, including any necessary amendment requests. No restrictions have been placed on the publication of the data and results will be shared with study participants.

All individuals have received the participant information sheet and provided informed consent prior to participation. Some individuals may find the questionnaires (e.g., MFQ) distressing. We will ensure that all participants are aware that their participation is voluntary and should they wish to withdraw at any time they will receive a list of local mental health resources and still receive compensation for their time. Participants will receive £30 compensation for their time for each phase, or £3 for each questionnaire if they choose to withdraw before completion of the study.

Self-injury and suicidality will be assessed in the MFQ and disclosure of such will be automatically flagged to the research team. The psychiatrists affiliated with the study will review participant responses to ascertain imminent risk of self-injury or other risks to the participant. If such risk is identified, then a follow up conversation will take place to further ascertain the participant's wellbeing. If the participant appears at high risk, they will be

For peer review only - http://bmjopen.bmj.com/site/about/guidelines.xhtml

advised to call the First Response Service (111 option 2) or attend their local Accident and Emergency department. If the risk is moderate, they will be advised to contact their General Practitioner for support.

All data will be anonymised and collected in REDCap and linked with a unique ID number to personally identifiable information stored in a secure, password encrypted database. Only REACT study team members will have access to the database and will follow procedures in accordance with the UK Data Protection Act 2018.

CURRENT STATUS

As of June 2020, 79 out of 92 eligible participants from the RAISE study have expressed interest in taking part and of those, all have completed the baseline questionnaires. No participants have withdrawn. We anticipate that all phases of the study will be completed by November 2020.

4. DISCUSSION

As a result of the public health emergency from COVID-19, individuals around the world are experiencing prolonged periods of social isolation and stress. This study will examine how baseline neuro-immune responses and emotion regulation capacity to acute stress in adolescents with CA impact mental health outcomes, emotional processing, social behaviour, physical health and perceived stress during and after this universal stressor, with the unique approach of leveraging data collected prior to its onset. We hypothesise that during acute stress in the laboratory, increased emotion regulation through increased central executive response and lower salience activity, and reduced baseline cortisol and cytokine levels, either through blunted responsivity or improved down regulation following stress, will be associated with greater mental and physical resilience and lower perceived stress in response to, and during, the societal changes associated with COVID-19. Utilising an integrative

For peer review only

framework we will examine social, psychological and biological resilience factors across the trajectory of the COVID-19 lockdown. This study aims to identify the mechanisms that contribute to adolescent resilience, identify which individuals may benefit from preventative strategies, and inform the development of novel interventions to increase resilience and prevent the development of mental health disorders after adversity.

DATA SHARING

Data will be available at the University of Cambridge Repository and able to access upon reasonable request.

AUTHOR CONTRIBUTIONS

AS assisted with study design, data collection and drafted the protocol manuscript. LML conceptualised and co-designed the study, oversaw data collection and revised the manuscript. ED, MRD, SO and ES assisted with study design and were instrumental to the set-up and/or data collection for the study. KI and MK assisted with study design. ALvH conceptualised and co-designed the study, obtained financial support, oversaw all study procedures, and reviewed and revised the manuscript. All authors approved the final manuscript and agree to be accountable for all aspects of the work presented.

FUNDING

The REACT study is funded by two grants from the Royal Society to Dr. Anne-Laura van Harmelen (RGF\EA\180029 & RGF\R1\180064). This work was further supported by a Royal Society Dorothy Hodgkin fellowship for Dr Anne-Laura van Harmelen (DH150176), a Wolfe Health fellowship for Dr. Laura Moreno-López, and a Gates Cambridge Scholarship (OPP1144) for Emma Soneson.

CONFLICTS OF INTEREST

For peer review only - http://bmjopen.bmj.com/site/about/guidelines.xhtml

The authors declare that they have no conflicts of interest associated with this project.

REFERENCES

1. Bao YP, Sun YK, Meng SQ, Shi J, Lu L. 2019-nCoV epidemic: address mental health care to empower society. Lancet. 2020;395(10224):E37-E8.

2. Cao WJ, Fang ZW, Hou GQ, Han M, Xu XR, Dong JX, et al. The psychological impact of the COVID-19 epidemic on college students in China. Psychiatry Research. 2020;287.

3. Holmes EA, O'Connor RC, Perry VH, Tracey I, Wessely S, Arseneault L, et al. Multidisciplinary research priorities for the COVID-19 pandemic: a call for action for mental health science. The lancet Psychiatry. 2020;7(6):547-60.

4. Brooks SK, Webster RK, Smith LE, Woodland L, Wessely S, Greenberg N, et al. The psychological impact of quarantine and how to reduce it: rapid review of the evidence. Lancet. 2020;395(10227):912-20.

5. Hampshire A, Hellyer P, Soreq E, Trender W, Mehta MA, Ioannidis K, et al. Dimensions and modulators of behavioural and mental-health changes during the Covid-19 pandemic: an N=343,017 study. 2020.

6. Chandan JS, Taylor J, Bradbury-Jones C, Nirantharakumar K, Kane E, Bandyopadhyay S. COVID-19: a public health approach to manage domestic violence is needed. Lancet Public Health. 2020;5(6):E309-E.

7. McLaughlin KA, Green JG, Gruber MJ, Sampson NA, Zaslavsky AM, Kessler RC. Childhood Adversities and Adult Psychiatric Disorders in the National Comorbidity Survey

For peer review only

For peer review only - http://bmjopen.bmj.com/site/about/guidelines.xhtml

Replication II Associations With Persistence of DSM-IV Disorders. Archives of General Psychiatry. 2010;67(2):124-32.

8. Kessler RC, Davis CG, Kendler KS. Childhood adversity and adult psychiatric disorder in the US National Comorbidity Survey. Psychological Medicine. 1997;27(5):1101-19.

9. Spinhoven P, Elzinga BM, Hovens J, Roelofs K, Zitman FG, van Oppen P, et al. The specificity of childhood adversities and negative life events across the life span to anxiety and depressive disorders. Journal of Affective Disorders. 2010;126(1-2):103-12.

10. Kalisch R, Baker DG, Basten U, Boks MP, Bonanno GA, Brummelman E, et al. The resilience framework as a strategy to combat stress-related disorders. Nature Human Behaviour. 2017;1(11):784-90.

11. Ioannidis K, Askelund AD, Kievit RA, Van Harmelen AL. The complex neurobiology of resilient functioning after childhood maltreatment. Bmc Medicine. 2020;18(1).

12. Moreno-Lopez L, Ioannidis K, Askelund AD, Smith AJ, Schueler K, Van Harmelen AL. The Resilient Emotional Brain: A Scoping Review of the Medial Prefrontal Cortex and Limbic Structure and Function in Resilient Adults With a History of Childhood Maltreatment. Biological Psychiatry-Cognitive Neuroscience and Neuroimaging. 2020;5(4):392-402.

13. Fritz J, de Graaff AM, Caisley H, van Harmelen AL, Wilkinson PO. A Systematic Review of Amenable Resilience Factors That Moderate and/or Mediate the Relationship Between Childhood Adversity and Mental Health in Young People. Frontiers in Psychiatry. 2018;9.

14. Liang L, Ren H, Cao R, Hu Y, Qin Z, Li C, et al. The Effect of COVID-19 on Youth Mental Health. Psychiatric Quarterly. 2020.

For peer review only

15. Li JB, Yang A, Dou K, Cheung RYM. Self-control moderates the association between perceived severity of the coronavirus disease 2019 (COVID-19) and mental health problems among the Chinese public. 2020.

16. Veer IM, Riepenhausen A, Zerban M, Wackerhagen C, Engen H, Puhlmann L, et al. Mental resilience in the Corona lockdown: First empirical insights from Europe. 2020.

17. Kalisch R, Muller MB, Tuscher O. A conceptual framework for the neurobiological study of resilience. Behavioral and Brain Sciences. 2015;38.

18. Surtees P, Wainwright N, Day N, Brayne C, Luben R, Khaw KT. Adverse experience in childhood as a developmental risk factor for altered immune status in adulthood. International Journal of Behavioral Medicine. 2003;10(3):251-68.

19. Marsland AL, Herbert TB, Muldoon MF, Bachen EA, Patterson S, Cohen S, et al. Lymphocyte subset redistribution during acute laboratory stress in young adults: Mediating effects of hemoconcentration. Health Psychology. 1997;16(4):341-8.

20. Nusslock R, Miller GE. Early-Life Adversity and Physical and Emotional Health Across the Lifespan: A Neuroimmune Network Hypothesis. Biological Psychiatry. 2016;80(1):23-32.

21. van Harmelen AL, Hauber K, Moor BG, Spinhoven P, Boon AE, Crone EA, et al. Childhood Emotional Maltreatment Severity Is Associated with Dorsal Medial Prefrontal Cortex Responsivity to Social Exclusion in Young Adults. Plos One. 2014;9(1).

22. Marsland AL, Walsh C, Lockwood K, John-Henderson NA. The effects of acute psychological stress on circulating and stimulated inflammatory markers: A systematic review and meta-analysis. Brain Behavior and Immunity. 2017;64:208-19.

23. Silverman MN, Sternberg EM. Glucocorticoid regulation of inflammation and its functional correlates: from HPA axis to glucocorticoid receptor dysfunction. Neuroimmunomodulation in Health and Disease I. 2012;1261:55-63.

For peer review only - http://bmjopen.bmj.com/site/about/guidelines.xhtml

24. Vecchiarelli HA, Gandhi CP, Gray JM, Morena M, Hassan KI, Hill MN. Divergent responses of inflammatory mediators within the amygdala and medial prefrontal cortex to acute psychological stress. Brain Behavior and Immunity. 2016;51:70-91.

25. Etkin A, Buchel C, Gross JJ. The neural bases of emotion regulation. Nature Reviews Neuroscience. 2015;16(11):693-+.

26. Elliott R. Executive functions and their disorders. British Medical Bulletin. 2003;65:49-59.

27. Gianaros PJ, Jennings JR, Sheu LK, Greer PJ, Kuller LH, Matthews KA. Prospective reports of chronic life stress predict decreased grey matter volume in the hippocampus. Neuroimage. 2007;35(2):795-803.

28. Slavich GM, Irwin MR. From Stress to Inflammation and Major Depressive Disorder: A Social Signal Transduction Theory of Depression. Psychological Bulletin. 2014;140(3):774-815.

29. Davidson RJ, Lewis DA, Alloy LB, Amaral DG, Bush G, Cohen JD, et al. Neural and Behavioral substrates of mood and mood regulation. Biological Psychiatry. 2002;52(6):478-502.

30. Danese A, Moffitt TE, Pariante CM, Ambler A, Poulton R, Caspi A. Elevated inflammation levels in depressed adults with a history of childhood maltreatment. Archives of General Psychiatry. 2008;65(4):409-16.

31. Khoury JE, Enlow MB, Plamondon A, Lyons-Ruth K. The association between adversity and hair cortisol levels in humans: A meta-analysis. Psychoneuroendocrinology. 2019;103:104-17.

32. Fogelman N, Canli T. Early life stress and cortisol: A meta-analysis. Hormones and Behavior. 2018;98:63-76.

For peer review only - http://bmjopen.bmj.com/site/about/guidelines.xhtml

33. Dedovic K, Renwick R, Mahani NK, Engert V, Lupien SJ, Pruessner JC. The Montreal Imaging Stress Task: using functional imaging to investigate the effects of perceiving and processing psychosocial stress in the human brain. Journal of Psychiatry & Neuroscience. 2005;30(5):319-25.

34. Baumeister D, Akhtar R, Ciufolini S, Pariante CM, Mondelli V. Childhood trauma and adulthood inflammation: a meta-analysis of peripheral C-reactive protein, interleukin-6 and tumour necrosis factor-alpha. Molecular Psychiatry. 2016;21(5):642-9.

35. Carpenter LL, Gawuga CE, Tyrka AR, Lee JK, Anderson GM, Price LH. Association between Plasma IL-6 Response to Acute Stress and Early-Life Adversity in Healthy Adults. Neuropsychopharmacology. 2010;35(13):2617-23.

36. Hartwell KJ, Maria M, Twal WO, Shaftman S, DeSantis SM, McRae-Clark AL, et al. Association of elevated cytokines with childhood adversity in a sample of healthy adults. Journal of Psychiatric Research. 2013;47(5):604-10.

37. Chamberlain SR, Cavanagh J, de Boer P, Mondelli V, Jones DNC, Drevets WC, et al. Treatment-resistant depression and peripheral C-reactive protein. British Journal of Psychiatry. 2019;214(1):11-9.

38. Ladouceur CD. COVID-19 Adolescent Symptom & Psychological Experience (CASPE)

Questionnaire. 2020.

39. Goodyer IM, Herbert J, Tamplin A, Secher SM, Pearson J. Short-term outcome of major depression .2. Life events, family dysfunction, and friendship difficulties as predictors of persistent disorder. Journal of the American Academy of Child and Adolescent Psychiatry. 1997;36(4):474-80.

For peer review only

40. Schat ACH, Kelloway EK, Desmarais S. The Physical Health Questionnaire (PHQ): Construct validation of a self-report scale of somatic symptoms. Journal of Occupational Health Psychology. 2005;10(4):363-81.

41. Angold A, Costello EJ, Messer SC, Pickles A, Winder F, Silver D. Development of a short questionnaire for use in epidemiological studies of depression in children and adolescents. International Journal of Methods in Psychiatric Research. 1995;5(4):237-49.

42. Daviss WB, Birmaher B, Melhem NA, Axelson DA, Michaels SM, Brent DA. Criterion validity of the Mood and Feelings Questionnaire for depressive episodes in clinic and non-clinic subjects. Journal of Child Psychology and Psychiatry. 2006;47(9):927-34.

43. Wood A, Kroll L, Moore A, Harrington R. PROPERTIES OF THE MOOD AND FEELINGS QUESTIONNAIRE IN ADOLESCENT PSYCHIATRIC OUTPATIENTS - A RESEARCH NOTE. Journal of Child Psychology and Psychiatry and Allied Disciplines. 1995;36(2):327-34.

44. Reynolds CR, Richmond BO. What I think and feel: A revised measure of children's manifest anxiety (Reprinted from Journal of Abnormal Child Psychology, vol 6, pg 271-280, 1978). Journal of Abnormal Child Psychology. 1997;25(1):15-20.

45. Tennant R, Hiller L, Fishwick R, Platt S, Joseph S, Weich S, et al. The Warwick-Edinburgh mental well-being scale (WEMWBS): development and UK validation. Health and Quality of Life Outcomes. 2007;5.

46. Goodman R. The strengths and difficulties questionnaire: A research note. Journal of Child Psychology and Psychiatry and Allied Disciplines. 1997;38(5):581-6.

47. Cohen S, Kamarck T, Mermelstein R. A GLOBAL MEASURE OF PERCEIVED STRESS. Journal of Health and Social Behavior. 1983;24(4):385-96.

For peer review only

48. Russell D, Peplau LA, Cutrona CE. THE REVISED UCLA LONELINESS SCALE - CONCURRENT AND DISCRIMINANT VALIDITY EVIDENCE. Journal of Personality and Social Psychology. 1980;39(3):472-80.

49. van Harmelen AL, Gibson JL, St Clair MC, Owens M, Brodbeck J, Dunn V, et al. Friendships and Family Support Reduce Subsequent Depressive Symptoms in At-Risk Adolescents. Plos One. 2016;11(5).

50. van Harmelen AL, Kievit RA, Ioannidis K, Neufeld S, Jones PB, Bullmore E, et al. Adolescent friendships predict later resilient functioning across psychosocial domains in a healthy community cohort. Psychological Medicine. 2017;47(13):2312-22.

51. McArdle JJ, Epstein D. LATENT GROWTH-CURVES WITHIN DEVELOPMENTAL STRUCTURAL EQUATION MODELS. Child Development. 1987;58(1):110-33.

52. Muthen BO, Curran PJ. General longitudinal modeling of individual differences in experimental designs: A latent variable framework for analysis and power estimation. Psychological Methods. 1997;2(4):371-402.

For peer review only

BMJ Open

The REACT study protocol: Resilience after the COVID-19 Threat (REACT) in adolescents.

Journal:	*BMJ Open*
Manuscript ID	bmjopen-2020-042824.R1
Article Type:	Protocol
Date Submitted by the Author:	22-Oct-2020
Complete List of Authors:	Smith, Alicia; University of Cambridge, Department of Psychiatry; University of Cambridge, MRC Cognition and Brain Sciences Unit<br>Moreno-López, Laura; University of Cambridge School of Clinical Medicine, Department of Psychiatry<br>Davidson, Eugenia; University of Cambridge, Department of Psychiatry; University of Exeter, College of Medicine and Health<br>Dauvermann, Maria; University of Cambridge, Department of Psychiatry; Massachusetts Institute of Technology, McGovern Institute for Brain Research<br>Orellana, Sofia; University of Cambridge, Department of Psychiatry<br>Soneson, Emma ; University of Cambridge Department of Psychiatry, Department of Psychiatry<br>Ioannidis, Konstantinos; University of Cambridge School of Clinical Medicine, Department of Psychiatry; Cambridgeshire and Peterborough NHS Foundation Trust<br>Kaser, Muzaffer; University of Cambridge School of Clinical Medicine; Cambridgeshire and Peterborough NHS Foundation Trust<br>van Harmelen, Anne-Laura; University of Cambridge School of Clinical Medicine, Department of Psychiatry; Leiden University, Education and Child Studies
**Primary Subject Heading**:	Mental health
Secondary Subject Heading:	Immunology (including allergy)
Keywords:	COVID-19, Neurobiology < NATURAL SCIENCE DISCIPLINES, PSYCHIATRY, Child & adolescent psychiatry < PSYCHIATRY

SCHOLARONE™
Manuscripts

I, the Submitting Author has the right to grant and does grant on behalf of all authors of the Work (as defined in the below author licence), an exclusive licence and/or a non-exclusive licence for contributions from authors who are: i) UK Crown employees; ii) where BMJ has agreed a CC-BY licence shall apply, and/or iii) in accordance with the terms applicable for US Federal Government officers or employees acting as part of their official duties; on a worldwide, perpetual, irrevocable, royalty-free basis to BMJ Publishing Group Ltd ("BMJ") its licensees and where the relevant Journal is co-owned by BMJ to the co-owners of the Journal, to publish the Work in this journal and any other BMJ products and to exploit all rights, as set out in our licence.

The Submitting Author accepts and understands that any supply made under these terms is made by BMJ to the Submitting Author unless you are acting as an employee on behalf of your employer or a postgraduate student of an affiliated institution which is paying any applicable article publishing charge ("APC") for Open Access articles. Where the Submitting Author wishes to make the Work available on an Open Access basis (and intends to pay the relevant APC), the terms of reuse of such Open Access shall be governed by a Creative Commons licence – details of these licences and which Creative Commons licence will apply to this Work are set out in our licence referred to above.

Other than as permitted in any relevant BMJ Author's Self Archiving Policies, I confirm this Work has not been accepted for publication elsewhere, is not being considered for publication elsewhere and does not duplicate material already published. I confirm all authors consent to publication of this Work and authorise the granting of this licence.

For peer review only

The REACT study protocol: Resilience after the COVID-19 Threat (REACT) in adolescents.

Alicia J. Smith[1,2*], Laura Moreno-López[3], Eugenia Davidson[4,5], Maria R. Dauvermann[6,7], Sofia Orellana[8], Emma Soneson[9], Konstantinos Ioannidis[10,11], Muzaffer Kaser[12,13] & Anne-Laura van Harmelen[14,15*]

*Corresponding authors

Word count = 4067
Abstract = 293

[1] Department of Psychiatry, University of Cambridge, Douglas House, 18b Trumpington Rd, Cambridge CB2 8AH, UK.
[2] MRC Cognition and Brain Sciences Unit, University of Cambridge, 15 Chaucer Road, Cambridge, CB2 7EF, UK. alicia.smith@mrc-cbu.cam.ac.uk
[3] Department of Psychiatry, University of Cambridge, Douglas House, 18b Trumpington Rd, Cambridge CB2 8AH, UK. lm618@medschl.cam.ac.uk
[4] Department of Psychiatry, University of Cambridge, Douglas House, 18b Trumpington Rd, Cambridge CB2 8AH, UK. ed555@medschl.cam.ac.uk
[5] College of Medicine and Health, University of Exeter, Stocker Rd, Exeter, EX4 4PY, UK. ed555@medschl.cam.ac.uk
[6] Department of Psychiatry, University of Cambridge, Douglas House, 18b Trumpington Rd, Cambridge CB2 8AH, UK. md874@medschl.cam.ac.uk
[7] McGovern Institute for Brain Research, Massachusetts Institute of Technology, Cambridge, USA. md874@medschl.cam.ac.uk
[8] Department of Psychiatry, University of Cambridge, Douglas House, 18b Trumpington Rd, Cambridge CB2 8AH, UK. sdco2@medschl.cam.ac.uk
[9] Department of Psychiatry, University of Cambridge, Douglas House, 18b Trumpington Rd, Cambridge CB2 8AH, UK. es703@medschl.cam.ac.uk
[10] Department of Psychiatry, University of Cambridge, Douglas House, 18b Trumpington Rd, Cambridge CB2 8AH, UK. konstantinos.ioannidis@cpft.nhs.uk
[11] Cambridgeshire and Peterborough NHS Foundation Trust, Fulbourn, UK. konstantinos.ioannidis@cpft.nhs.uk
[12] Department of Psychiatry, University of Cambridge, Douglas House, 18b Trumpington Rd, Cambridge CB2 8AH, UK. mk708@medschl.cam.ac.uk
[13] Cambridgeshire and Peterborough NHS Foundation Trust, Fulbourn, UK. mk708@medschl.cam.ac.uk
[14] Department of Psychiatry, University of Cambridge, Douglas House, 18b Trumpington Rd, Cambridge CB2 8AH, UK. Av391@medschl.cam.ac.uk
[15] Institute of Education and Child Studies, Leiden University, Pieter de la Court building, 4th floor Wassenaarseweg 52, 2333 AK Leiden. a.van.harmelen@fsw.leidenuniv.nl

For peer review only

ABSTRACT

Introduction: Coronavirus disease 2019 (COVID-19) related social isolation and stress may have significant mental health effects, including post-traumatic stress, anxiety and depression. These effects are thought to disproportionally affect populations at risk for psychopathology, such as youth with a history of childhood adversity (CA). Therefore, examining which factors may buffer the impact of COVID-19 related stress and isolation in vulnerable youth is critical. The Resilience after COVID-19 Threat (REACT) study assesses whether emotion regulation capacity, inflammation and neuroimmune responses to stress induced in the laboratory prior to the pandemic predict responses to COVID-19 related social isolation and stress in adolescents with CA. We aim to elucidate the mechanisms that enable vulnerable youth to maintain or regain good mental health when confronted with COVID-19. **Methods and analysis**: We recruited 79 adolescents aged 16-26 with CA experiences from the Resilience after Individual Stress Exposure (RAISE) study in which we assessed emotion regulation, neural and immune stress responses to an acute stress task. Our sample completed questionnaires at the start of the UK lockdown ('baseline'; April 2020) and three (July 2020) and six months later (October 2020) providing crucial longitudinal information across phases of the pandemic progression and government response. The questionnaires assess 1) mental health, 2) number and severity of life events, 3) physical health, 4) stress perception, and 5) loneliness and friendship support. We will use multilevel modelling to examine whether individual differences at baseline are associated with responses to COVID-19 related social isolation and stress. **Ethics and dissemination:** This study has been approved by the Cambridge Psychology Research Ethics Committee (PRE.2020.037). Results of the REACT study will be disseminated in publications in scientific peer reviewed journals, presentations at scientific conferences and meetings, publications and presentations for the general public, and through social media.

For peer review only - http://bmjopen.bmj.com/site/about/guidelines.xhtml

Key words: Resilience; Stress; COVID-19; Childhood adversity; Maltreatment; Adolescence

For peer review only

For peer review only - http://bmjopen.bmj.com/site/about/guidelines.xhtml

ARTICLE SUMMARY

STRENGTHS AND LIMITATIONS

- Utilising an integrated bio-psycho-social design, we will be able to examine the relations and interrelations of emotion regulation, neural and immune responses in predicting health and wellbeing across the trajectory of the COVID-19 lockdown.
- Due to the prospective design, we will be able to examine intra-individual *changes* in wellbeing and stress perceptions across the COVID-19 pandemic.
- Due to deep neuro-immune phenotyping prior to COVID-19, we will be able to examine whether individual differences in neuro-immune factors predict wellbeing and stress perception in response to COVID-19.
- This study relies on retrospective self-report of childhood adversity which may be subject to biased recall.
- Due to the ongoing nature of the pandemic and fluctuations in lockdown restrictions, it may not be possible for us to gauge full recovery from the stress of the pandemic using our current timeline of assessments. For this reason, a fourth wave may be added at a later stage, depending on funding availability.

For peer review only

For peer review only - http://bmjopen.bmj.com/site/about/guidelines.xhtml

1. INTRODUCTION

The recent outbreak of a severe acute respiratory syndrome caused by the Coronavirus disease 2019 (COVID-19) originating in China has escalated globally (1). Governments have imposed nation-wide measures of disease containment and urged their citizens to practice social distancing and isolation. As a consequence, millions of people worldwide are currently experiencing unprecedented periods of social isolation and stress (2). Consequences of the social isolation and COVID-related stress will likely result in increased rates of depression, acute and post-traumatic stress and clinical anxiety, especially in already vulnerable populations (3, 4) and in demographically mediated ways (5).

Negative impact of the current pandemic is thought to disproportionally affect populations already at risk for psychopathology, such as youth with a history of childhood adversity (CA) (3, 6). CA, comprising emotional, sexual and/or physical abuse, emotional and/or physical neglect, marital distress/conflict, parental mental health problems and/or parental alcohol dependence, violence and/or aggressive behaviour prior to the age of 16, is one of the strongest predictors of poor mental health and wellbeing in adulthood (7-9).

Despite evidence of the detrimental effects of CA on mental health, a significant proportion of individuals show little or no long-term negative sequelae; they display 'resilience' (10). Currently, our understanding of *resilience* has been mostly limited to studying outcome after individual-level trauma or commonly experienced catastrophes such as terror attacks or natural disasters. So far, studies examining resilience have either focussed on [1] a trait like *capacity* that precedes adversity, [2] a dynamic *process* that unfolds during and after adversity, or [3] an *outcome* following adversity. Our recent resilience framework (10) combines these viewpoints and describes that *resilience* is the dynamic process of positive adaptation to stress, which is aided by resilience factors (traits and states; e.g. genetic

For peer review only - http://bmjopen.bmj.com/site/about/guidelines.xhtml

factors, hormonal levels, brain anatomy, social support), and can be measured in the aftermath of stress in the form of resilient functioning. Resilience in the context of CA, where the stressor has already taken place in childhood, can be examined through the examination of resilient functioning. Such resilient functioning after CA refers to functioning across a range of relevant domains (thoughts, feelings, mood, behaviour, academic ability) that is better than others with similar experiences. A detailed account of this quantification of resilient functioning, and the benefits and drawbacks of this method can be found in Ioannidis et al., (2020) (11).

The psychological impact of the current pandemic on the general public and modifiable resilience factors that may explain the individual differences in young people's responses have been identified. In China, a cross-sectional study of young people aged 14-35 found that 40.4% of the sample reported having psychological problems and 14.4% presented post-traumatic stress disorder (PTSD) symptoms two weeks after the outbreak of COVID-19 (12). Recent work by Li et al. (13) on 4607 Chinese citizens aged 16 years and over suggested that individuals' cognitive appraisals and perceived severity of the COVID-19 threat was predictive of negative emotional and behavioural responses. Similarly, data from Europe has revealed a positive association between resilience to stress exposure during the COVID-19 lockdown and positive appraisal style in a sample of N=5000 adults (14). In a large UK sample, personality traits of compassion, conscientiousness, perfectionism, and extravertedness were associated with experiencing a more positive global (psycho-socio-economic) impact of the COVID-19 pandemic (5). In sum, emotion regulation is thought to be a critical resilience factor (15).

It has been reported that adversity experienced in childhood has potential detrimental effects on emotion regulation capacity and stress regulation through internal biological processes, such as altered inflammatory processes (i.e. changes in circulating lymphocyte

For peer review only - http://bmjopen.bmj.com/site/about/guidelines.xhtml

count and proinflammatory markers) and neurobiological mechanisms (i.e. increased central executive response and lower salience network activity) (16-20). During exposure to stress, the body releases pro-inflammatory markers including high sensitivity C-reactive protein (hs-CRP) and cytokines, including interleukin (IL)-1β, IL-6 and tumor necrosis factor (TNF)-α (21). These cytokines play a key role in acute stress reactivity by activating the hypothalamic-pituitary-adrenal (HPA) axis to release glucocorticoids such as cortisol (22). In turn, cortisol suppresses the release of cytokines, playing an important anti-inflammatory role in acute stress recovery. While the priming on the immune system following acute stress likely represents an adaptive response, research suggests that acute stress causes an upregulation of inflammatory mediators in circulation and in the amygdala and a decrease in inflammatory mediators in the medial prefrontal cortex (mPFC) (23) – regions involved in executive functioning and emotion regulation (24, 25). The elevated release of proinflammatory markers in response to a chronic stressor, such as CA, has been associated with both structural and functional alterations in these brain regions (26). Furthermore, reduced volumes of the amygdala and the mPFC, as well as increased/decreased activation of these regions during executive and affective control tasks may in turn confer vulnerability to mood disorders such as depression and/or anxiety (27-29).

Research is required to understand how interrelated resilience factors, ranging from ‘bottom-up’ polygenetic influences to ‘top-down’ social environmental factors, can facilitate resilient functioning to new stress, such as COVID-19, after CA. The HPA axis, controlling stress reactions and immune functioning, is one system that inextricably intertwines with brain structure and function and may be linked to vulnerable or resilient responses to COVID-19. In the context of CA, cortisol responses to awakening and acute stress response differ for patients with and without psychopathology (30). Results of a recent meta-analysis revealed that in a healthy population, CA was associated with increased cortisol awakening

For peer review only - http://bmjopen.bmj.com/site/about/guidelines.xhtml

response and lower baseline cortisol response (31). In the ongoing Resilience after Individual Stress Exposure (RAISE) study we investigated how the HPA axis and immune system interrelate with brain structure and function, and social environment to facilitate resilient functioning by measuring emotion regulation capacity and stress responses to a well-validated functional magnetic resonance imaging (fMRI) task, the Montreal imaging stress task (MIST; (32)), in individuals with CA. In the current study, we have the unique opportunity to leverage the RAISE data to investigate how individual differences in emotion regulation and stress responses to previous laboratory-based stressors predicts individual differences in response to COVID-19 stress and isolation. By assessing individuals' responses before, during and after a universally experienced stressor, the REACT study will enable us to integrate and examine social, psychological and biological resilience factors across the trajectory and to predict resilience during stress and/or resilient functioning outcomes.

OBJECTIVES AND HYPOTHESIS

The aim of this study is to examine whether and how social support, emotion regulation capacity, baseline inflammation, and/or neuroimmune responses to laboratory-induced acute stress in adolescents predicts: 1) perceived stress, 2) emotional processing, 3) social behaviour, 4) general mental health, and 5) physical health across the trajectory of the COVID-19 restrictions and restructuring.

We hypothesise that social support, resilient emotion regulation and/or stress responses to previous laboratory-based stressors (both in the brain (i.e. increased central executive response and lower salience network activity) and in the periphery (i.e. lower levels of cortisol, IL-1b, IL-6, TNF-α and hs-CRP)) will be associated with greater resilient functioning as well as better mental and physical health and wellbeing (lower perceived

For peer review only - http://bmjopen.bmj.com/site/about/guidelines.xhtml

stress, lower loneliness and increased perception of peer support) across the trajectory of the COVID-19 pandemic (11, 19, 29, 33-36).

2. METHODS AND ANALYSIS

2.1. Study design

All participants have provided written informed consent to complete a set of eleven questionnaires at three time points: at baseline (April 2020), 3 months (July 2020) and 6 months (October 2020) thereafter. By distributing baseline questionnaires in April, a few weeks after the initial lockdown restrictions in the UK were imposed, the social restrictions serve as a timeline for us to measure the wider impact of the pandemic on society. Questionnaires sent at three and six months thereafter will measure responses as these restrictions are gradually eased. Beyond government measures, the COVID-19 pandemic has a pervasive impact on people's lives due to a substantial and reactive restructuring of society on multiple levels of ecology. The follow-up questionnaires will provide crucial longitudinal measurements, in the context of the impending socio-ecological change, following the phases of the pandemic progression and government response.

2.2. Patient and Public Involvement (PPI)

Patient and Public Involvement (PPI) was conducted prior to the RAISE study and feedback from the panel was utilised to improve the quality, relevance and impact of the REACT study. A group of 3 adolescents participated in the lived experience advisory group to assess our project and the materials we used to recruit, such as the participant information sheet and consent forms. As a result of this feedback, we made the following changes: (1) we have included a risk protocol in the case that a participant becomes distressed during the completion of the questionnaires, (2) within the questionnaires, we have added an evaluation of self-harm and suicidality, (3) we have increased the payment for the completion of the

For peer review only - http://bmjopen.bmj.com/site/about/guidelines.xhtml

study to account for the time burden of the questionnaires, and (4) we have modified the participant information sheet in accordance with their suggestions.

2.3. Participants and recruitment

Participants were recruited from the ongoing RAISE study carried out in the Department of Psychiatry at the University of Cambridge, which involves the completion of an online assessment to assess psychological functioning, emotion regulation capacity and early life experiences as well as an in-unit assessment at Addenbrooke's Hospital to assess neural and immune responses to laboratory-induced acute stress. We contacted 92 individuals from the RAISE Study, of which 79 participants expressed an interest in taking part in REACT and received the first set of questionnaires in April 2020.

Inclusion criteria for the RAISE study were: aged 16-26 years inclusive, able and willing to give informed consent, able to speak, write, and understand English, body mass index (BMI) between 18.5 and 29.9 kg/m2, experienced CA (emotional, sexual and/or physical abuse, emotional and/or physical neglect, marital distress/conflict, parental mental health problems and/or parental alcohol dependence, violence and/or aggressive behaviour before the age of 16). Exclusion criteria for the RAISE study were: alcohol or substance use disorder within the past six months, current disorders likely to compromise the interpretation of the data (e.g. immunological disorders, cardiovascular disorders, endocrine and autoimmune disorders, malignancies or infections, or any other condition to be determined by the principal investigator or delegate), current medication likely to compromise the interpretation of immunological data (including, but not limited to, corticosteroids or any other substance to be determined by the principal investigator or delegate).

Participants for the RAISE study were recruited in Cambridge, UK, through advertisements in the general community (e.g., posters in hospitals, colleges, coffee shops)

For peer review only - http://bmjopen.bmj.com/site/about/guidelines.xhtml

and on social media platforms (e.g., Facebook and Twitter advertisements). We also recruited from an existing database of participants who had previously taken part in the NeuroScience in Psychiatry Network (NSPN; N=2389) and had consented to being contacted for future studies.

2.4. Study procedure

All RAISE study participants who consented to being re-contacted for future studies were approached via email and provided with the participant information sheet. All interested participants were asked to complete online assessments at three time points and were encouraged to contact the study team with any questions that they had.

Participants who expressed interest in taking part received an email with the secure links and instructions to complete the first online consent form and set of questionnaires. The links to the baseline questionnaires were sent at the beginning of April. The same questionnaires and computer tasks will be sent three and six months thereafter to follow-up any changes experienced by the participant.

All three assessments (baseline (April 2020), three month (July 2020) and six month follow-ups (October 2020)) will include the following self-report questionnaires:

Life-Events Questionnaire (37). Number and severity of negative events experienced will be assessed with the Life Events Questionnaire (LEQ). Participants will rate 13 major life events which may have occurred during the preceding 18-month period including changes in school, illness, moving house, deaths and friendship difficulties. If affected by the event, respondents are asked to detail what happened and rate it on a scale of 1='very pleasant/happy' to 5='very unpleasant/sad/painful'. A higher score indicates a greater number of adverse life events experienced in the 18 months prior to the assessment.

For peer review only - http://bmjopen.bmj.com/site/about/guidelines.xhtml

Physical Health Questionnaire (PHQ) (38). Physical health will be assessed using a 14-item questionnaire that covers four areas of general physical health and areas that are often affected by stress (both acute and chronic), such as gastrointestinal problems, headaches, sleep disturbances, and respiratory illness. Higher scores indicates greater somatic health.

The Mood and Feelings Questionnaire (39). The Mood and Feelings Questionnaire (MFQ) is a 33-item instrument that was developed to assess mental health and wellbeing from two weeks prior and up to the date of the assessment. The MFQ has shown prognostic validity in both clinical and non-clinical samples (40, 41). Higher sum scores indicate higher levels of depressive symptoms.

The Revised Children's Manifest Anxiety Scale (42). The Revised Children's Manifest Anxiety Scale (RCMAS) is a 37-item instrument that assesses physiological anxiety, worry/oversensitivity, social concerns/concentration, and total anxiety score. A high score indicates higher levels of anxiety.

Warwick-Edinburgh Mental Wellbeing Scale (43). The Warwick-Edinburgh Mental Wellbeing Scale (WEMWBS) assesses responses to 14 positively worded statements to quantify mental wellbeing in the general population. Each item is graded by the respondent from 'none of the time' to 'all of the time'. Higher scores indicate greater wellbeing.

Strengths and Difficulties Questionnaire YR1 (44). The Strengths and Difficulties Questionnaire (SDQ) is a 25-item self-report measure, which is divided into five subscales. Four of the subscales measure difficulties and one subscale measures strengths, a prosocial behaviour. The difficulties subscales assess emotional symptoms (internalising symptoms), conduct problems, hyperactivity/inattention and peer relationship problems. The measure has been shown to have good psychometric properties in the age group that will be recruited for the current study (Cronbach's α of 0.80), as well as good sensitivity, specificity and

For peer review only

prospective utility. The total difficulties score is calculated by summing the scores of the four difficulties subscales. A high score indicates higher risk of clinically significant problems and can be used to identify likely cases of mental health problems. Conversely, a low score on the prosocial behaviour subscale indicates higher risk of clinically significant problems.

Cambridge friendship questionnaire. The Cambridge friendship questionnaire (CFQ) is an 8-item questionnaire that assesses the quality and number of friendships. The CFQ has good measurement invariance and external validity, and has demonstrated ecological validity across two samples (45, 46). Higher scores indicate better perceived overall quality of friendships.

Social isolation will be assessed using the *Perceived Stress Scale* and the *Revised UCLA Loneliness Scale*:

Perceived Stress Scale (47). The Perceived Stress Scale (PSS) is the most widely used psychological instrument for measuring the perception of stress. It is a 10-item measure used to assess the degree to which situations in one's life are appraised as stressful. Items are designed to assess how unpredictable, uncontrollable, and overloaded respondents find their lives. The scale also includes several direct queries about current levels of experienced stress. A higher sum score indicates higher perceived stress.

Revised UCLA Loneliness Scale (48). The Revised UCLA Loneliness Scale (R-UCLA) is a 20-item scale designed to measure subjective feelings of loneliness and social isolation. Participants rate each item on a scale from 1 (Never) to 4 (Often). Higher scores indicate a greater degree of loneliness.

COVID-19 related stress will be recorded using the *COVID-19 Adolescent Symptom & Psychological Experience Questionnaire:*

For peer review only - http://bmjopen.bmj.com/site/about/guidelines.xhtml

For peer review only

COVID-19 Adolescent Symptom & Psychological Experience Questionnaire (49). Emotional, cognitive and social experiences related to COVID-19 will be addressed using the COVID-19 Adolescent Symptom & Psychological Experience Questionnaire (CASPE). The CASPE forms part of the research tracker and facilitator for Assessment of COVID-19 Experiences (ACE) for Adolescents developed by scientists at the University of Oregon (https://doi.org/10.17605/OSF.IO/PY7VG). The CASPE has a total of 38 items distributed in four major categories: 1) experience related to COVID-19 and symptoms, 2) emotional experience, 3) cognitive experience, and 4) social experience.

2.5. Phenotyping

Up to 30 mL of blood were obtained from each participant during the in-unit assessment for the RAISE Study. Measurements of cortisol, blood cytokines and immune cells in response to acute stress will be analysed using Partial Least Squares (PLS) to determine whether or not resilient adolescents can be distinguished based on immune patterns. By deep neuro-immune-phenotyping we will be able to examine whether individual differences in neuro-immune factors predict wellbeing and stress perception in response to COVID-19.

2.6. Statistical analysis plan

Responses during and after COVID-19 will be assessed using growth modelling analyses, a statistical technique implemented within a Structural Equation Modelling (SEM) framework, which allows for the estimation of subject-specific trajectories of change across time for a given set of variables of interest (50, 51). Specifically, implementing growth modelling analyses, we will assess whether individual differences in emotion regulation capacity, and/or neural and immune responses to stress in the laboratory at baseline are predictive of individual health and wellbeing at baseline (intercepts) or trajectories of change before, during and after COVID-19 (slopes). Particularly, we will estimate intercepts and slopes for

For peer review only - http://bmjopen.bmj.com/site/about/guidelines.xhtml

mental and physical health and wellbeing, perceived stress, loneliness and friendship support. In these models, we will add differences in social distancing behaviours and negative life events experienced as covariates, in addition to age, sex and BMI. We will then input these parameters in regression analyses in order to determine whether they are associated with individual differences in emotion regulation capacity and/or neural and immune responses to stress in the laboratory. The analysis of emotion regulation capacity, and/or neural and immune responses to stress in the laboratory at baseline in the RAISE study have been explained in detail in the RAISE study protocol (52). In short, principle component analyses (PCA) will be utilized to derive a factor score for endocrine and inflammatory markers at the different time points (before and after the exposition to a stress task) and imaging data will be analysed using well-established methods for quantification of structural and functional parameters.

ETHICS AND RISKS

The study has been approved by the Cambridge Psychology Research Ethics Committee (PRE.2020.037). Ethical guidelines will be adhered to throughout the study, including any necessary amendment requests. No restrictions have been placed on the publication of the data and results will be shared with study participants.

All individuals have received the participant information sheet and provided informed consent prior to participation. Some individuals may find the questionnaires (e.g., MFQ) distressing. We will ensure that all participants are aware that their participation is voluntary and should they wish to withdraw at any time they will receive a list of local mental health resources and still receive compensation for their time. Participants will receive £30 compensation for their time for each phase, or £3 for each questionnaire if they choose to withdraw before completion of the study.

For peer review only - http://bmjopen.bmj.com/site/about/guidelines.xhtml

Self-injury and suicidality will be assessed in the MFQ and disclosure of such will be automatically flagged to the research team. The psychiatrists affiliated with the study will review participant responses to ascertain imminent risk of self-injury or other risks to the participant. If such risk is identified, then a follow up conversation will take place to further ascertain the participant's wellbeing. If the participant appears at high risk, they will be advised to call the First Response Service (111 option 2) or attend their local Accident and Emergency department. If the risk is moderate, they will be advised to contact their General Practitioner for support.

All data will be anonymised and collected in REDCap and linked with a unique ID number to personally identifiable information stored in a secure, password encrypted database. Only REACT study team members will have access to the database and will follow procedures in accordance with the UK Data Protection Act 2018.

3.0 DISSEMINATION

The results of the REACT study will be disseminated through (1) publications in scientific peer reviewed journals, (2) presentations on relevant scientific conferences and meetings, (3) publications and presentations for the general public and (4) through social media.

1) **Publications in scientific peer reviewed journals**. Given the importance of this research subject we expect to disseminate the results through publications in high impact journals such as Lancet Psychiatry, JAMA Psychiatry, Molecular Psychiatry, Biological Psychiatry and E-life.

2) **Presentations on relevant scientific conferences and meetings**. The team will present the results of the study in the form of symposia at conferences and scientific meetings across the world. Anticipated conferences of interest are the Society of Biological Psychiatry conference, FLUX, Society for Neuroscience, and the Organization for Human Brain Mapping meeting.

For peer review only - http://bmjopen.bmj.com/site/about/guidelines.xhtml

3) **Publications and presentations for the general public**. The team is passionate about public engagement. Our team have written many publications for the general public and organised the conference 'No nurture, No Chance; Resilience after trauma', which was sold out to 150 members from the general public. In addition, both Cambridge (and nearby London) offer many opportunities for scientific outreach such as the Cambridge Science Festival, Pint of Science (Cambridge) and the London Science Festival.

4) **Social media presence**. The team has excellent experience with media engagement. Our team is active on social media (twitter), and frequently uses twitter to promote research. Furthermore, the team has excellent lines with established media to promote research. For instance, our team have given many interviews in the traditional media as well as with the 'naked scientist' podcast, and our work has been featured in the Guardian, in blogs and in podcasts.

CURRENT STATUS

As of October 2020, 79 participants from the RAISE study expressed an interest in taking part in REACT, and completed the baseline questionnaires. From this sample, 77 participants completed the second phase of questionnaires. We anticipate that all phases of the study will be completed by November 2020.

4.0 DISCUSSION

As a result of the public health emergency from COVID-19, individuals around the world are experiencing prolonged periods of social isolation and stress. This study will examine how baseline neuro-immune responses and emotion regulation capacity to acute stress in adolescents with CA impact mental health outcomes, emotional processing, social behaviour, physical health and perceived stress during and after this universal stressor, with the unique approach of leveraging data collected prior to its onset. We hypothesise that during acute

For peer review only - http://bmjopen.bmj.com/site/about/guidelines.xhtml

stress in the laboratory, increased emotion regulation through increased central executive response and lower salience activity, and reduced baseline cortisol and cytokine levels, either through blunted responsivity or improved down regulation following stress, will be associated with greater mental and physical resilience and lower perceived stress in response to, and during, the societal changes associated with COVID-19. Utilising an integrative framework we will examine social, psychological and biological resilience factors across the trajectory of the COVID-19 lockdown. This study aims to characterise the neurobiological mechanisms that contribute to adolescent resilient functioning. We hope that our study will provide important insights for intervention studies, for instance the development of strategies encompassing psychological, cognitive behavioural, somatic or psychopharmacological therapies. Furthermore, our findings may inform the development of current or novel interventions to increase resilience by preventing the development of mental health disorders after adversity.

DATA SHARING

Data will be available at the University of Cambridge Repository and able to access upon reasonable request.

AUTHOR CONTRIBUTIONS

AS assisted with study design, data collection, and drafted and revised the protocol manuscript. LML conceptualised and co-designed the study, oversaw data collection and revised the manuscript. ED, MRD, SO and ES assisted with study design and were instrumental to the set-up and/or data collection for the study. KI and MK assisted with study design. ALvH conceptualised and co-designed the study, obtained financial support, oversaw all study procedures, and reviewed and revised the manuscript. All authors approved the final manuscript and agree to be accountable for all aspects of the work presented.

For peer review only - http://bmjopen.bmj.com/site/about/guidelines.xhtml

For peer review only

FUNDING

The REACT study is funded by two grants from the Royal Society to Dr. Anne-Laura van Harmelen (RGF\EA\180029 & RGF\R1\180064). This work was further supported by a Royal Society Dorothy Hodgkin fellowship for Dr Anne-Laura van Harmelen (DH150176), a Wolfe Health fellowship for Dr. Laura Moreno-López, and a Gates Cambridge Scholarship (OPP1144) for Emma Soneson.

CONFLICTS OF INTEREST

The authors declare that they have no conflicts of interest associated with this project.

REFERENCES

1. Bao YP, Sun YK, Meng SQ, Shi J, Lu L. 2019-nCoV epidemic: address mental health care to empower society. Lancet. 2020;395(10224):E37-E8.

2. Cao WJ, Fang ZW, Hou GQ, Han M, Xu XR, Dong JX, et al. The psychological impact of the COVID-19 epidemic on college students in China. Psychiatry Research. 2020;287.

3. Holmes EA, O'Connor RC, Perry VH, Tracey I, Wessely S, Arseneault L, et al. Multidisciplinary research priorities for the COVID-19 pandemic: a call for action for mental health science. The lancet Psychiatry. 2020;7(6):547-60.

4. Brooks SK, Webster RK, Smith LE, Woodland L, Wessely S, Greenberg N, et al. The psychological impact of quarantine and how to reduce it: rapid review of the evidence. Lancet. 2020;395(10227):912-20.

5. Hampshire A, Hellyer P, Soreq E, Trender W, Mehta MA, Ioannidis K, et al. Dimensions and modulators of behavioural and mental-health changes during the Covid-19 pandemic: an N=343,017 study. 2020.

For peer review only - http://bmjopen.bmj.com/site/about/guidelines.xhtml

6. Chandan JS, Taylor J, Bradbury-Jones C, Nirantharakumar K, Kane E, Bandyopadhyay S. COVID-19: a public health approach to manage domestic violence is needed. Lancet Public Health. 2020;5(6):E309-E.

7. McLaughlin KA, Green JG, Gruber MJ, Sampson NA, Zaslavsky AM, Kessler RC. Childhood Adversities and Adult Psychiatric Disorders in the National Comorbidity Survey Replication II Associations With Persistence of DSM-IV Disorders. Archives of General Psychiatry. 2010;67(2):124-32.

8. Kessler RC, Davis CG, Kendler KS. Childhood adversity and adult psychiatric disorder in the US National Comorbidity Survey. Psychological Medicine. 1997;27(5):1101-19.

9. Spinhoven P, Elzinga BM, Hovens J, Roelofs K, Zitman FG, van Oppen P, et al. The specificity of childhood adversities and negative life events across the life span to anxiety and depressive disorders. Journal of Affective Disorders. 2010;126(1-2):103-12.

10. Kalisch R, Baker DG, Basten U, Boks MP, Bonanno GA, Brummelman E, et al. The resilience framework as a strategy to combat stress-related disorders. Nature Human Behaviour. 2017;1(11):784-90.

11. Ioannidis K, Askelund AD, Kievit RA, Van Harmelen AL. The complex neurobiology of resilient functioning after childhood maltreatment. Bmc Medicine. 2020;18(1).

12. Liang L, Ren H, Cao R, Hu Y, Qin Z, Li C, et al. The Effect of COVID-19 on Youth Mental Health. Psychiatric Quarterly. 2020.

13. Li JB, Yang A, Dou K, Cheung RYM. Self-control moderates the association between perceived severity of the coronavirus disease 2019 (COVID-19) and mental health problems among the Chinese public. 2020.

For peer review only

For peer review only - http://bmjopen.bmj.com/site/about/guidelines.xhtml

14. Veer IM, Riepenhausen A, Zerban M, Wackerhagen C, Engen H, Puhlmann L, et al. Mental resilience in the Corona lockdown: First empirical insights from Europe. 2020.

15. Kalisch R, Muller MB, Tuscher O. A conceptual framework for the neurobiological study of resilience. Behavioral and Brain Sciences. 2015;38.

16. Surtees P, Wainwright N, Day N, Brayne C, Luben R, Khaw KT. Adverse experience in childhood as a developmental risk factor for altered immune status in adulthood. International Journal of Behavioral Medicine. 2003;10(3):251-68.

17. Marsland AL, Herbert TB, Muldoon MF, Bachen EA, Patterson S, Cohen S, et al. Lymphocyte subset redistribution during acute laboratory stress in young adults: Mediating effects of hemoconcentration. Health Psychology. 1997;16(4):341-8.

18. Nusslock R, Miller GE. Early-Life Adversity and Physical and Emotional Health Across the Lifespan: A Neuroimmune Network Hypothesis. Biological Psychiatry. 2016;80(1):23-32.

19. Moreno-Lopez L, Ioannidis K, Askelund AD, Smith AJ, Schueler K, Van Harmelen AL. The Resilient Emotional Brain: A Scoping Review of the Medial Prefrontal Cortex and Limbic Structure and Function in Resilient Adults With a History of Childhood Maltreatment. Biological Psychiatry-Cognitive Neuroscience and Neuroimaging. 2020;5(4):392-402.

20. van Harmelen AL, Hauber K, Moor BG, Spinhoven P, Boon AE, Crone EA, et al. Childhood Emotional Maltreatment Severity Is Associated with Dorsal Medial Prefrontal Cortex Responsivity to Social Exclusion in Young Adults. Plos One. 2014;9(1).

21. Marsland AL, Walsh C, Lockwood K, John-Henderson NA. The effects of acute psychological stress on circulating and stimulated inflammatory markers: A systematic review and meta-analysis. Brain Behavior and Immunity. 2017;64:208-19.

For peer review only

22. Silverman MN, Sternberg EM. Glucocorticoid regulation of inflammation and its functional correlates: from HPA axis to glucocorticoid receptor dysfunction. Neuroimmunomodulation in Health and Disease I. 2012;1261:55-63.

23. Vecchiarelli HA, Gandhi CP, Gray JM, Morena M, Hassan KI, Hill MN. Divergent responses of inflammatory mediators within the amygdala and medial prefrontal cortex to acute psychological stress. Brain Behavior and Immunity. 2016;51:70-91.

24. Etkin A, Buchel C, Gross JJ. The neural bases of emotion regulation. Nature Reviews Neuroscience. 2015;16(11):693-+.

25. Elliott R. Executive functions and their disorders. British Medical Bulletin. 2003;65:49-59.

26. Gianaros PJ, Jennings JR, Sheu LK, Greer PJ, Kuller LH, Matthews KA. Prospective reports of chronic life stress predict decreased grey matter volume in the hippocampus. Neuroimage. 2007;35(2):795-803.

27. Slavich GM, Irwin MR. From Stress to Inflammation and Major Depressive Disorder: A Social Signal Transduction Theory of Depression. Psychological Bulletin. 2014;140(3):774-815.

28. Davidson RJ, Lewis DA, Alloy LB, Amaral DG, Bush G, Cohen JD, et al. Neural and Behavioral substrates of mood and mood regulation. Biological Psychiatry. 2002;52(6):478-502.

29. Danese A, Moffitt TE, Pariante CM, Ambler A, Poulton R, Caspi A. Elevated inflammation levels in depressed adults with a history of childhood maltreatment. Archives of General Psychiatry. 2008;65(4):409-16.

30. Khoury JE, Enlow MB, Plamondon A, Lyons-Ruth K. The association between adversity and hair cortisol levels in humans: A meta-analysis. Psychoneuroendocrinology. 2019;103:104-17.

For peer review only - http://bmjopen.bmj.com/site/about/guidelines.xhtml

31. Fogelman N, Canli T. Early life stress and cortisol: A meta-analysis. Hormones and Behavior. 2018;98:63-76.

32. Dedovic K, Renwick R, Mahani NK, Engert V, Lupien SJ, Pruessner JC. The Montreal Imaging Stress Task: using functional imaging to investigate the effects of perceiving and processing psychosocial stress in the human brain. Journal of Psychiatry & Neuroscience. 2005;30(5):319-25.

33. Baumeister D, Akhtar R, Ciufolini S, Pariante CM, Mondelli V. Childhood trauma and adulthood inflammation: a meta-analysis of peripheral C-reactive protein, interleukin-6 and tumour necrosis factor-alpha. Molecular Psychiatry. 2016;21(5):642-9.

34. Carpenter LL, Gawuga CE, Tyrka AR, Lee JK, Anderson GM, Price LH. Association between Plasma IL-6 Response to Acute Stress and Early-Life Adversity in Healthy Adults. Neuropsychopharmacology. 2010;35(13):2617-23.

35. Hartwell KJ, Maria M, Twal WO, Shaftman S, DeSantis SM, McRae-Clark AL, et al. Association of elevated cytokines with childhood adversity in a sample of healthy adults. Journal of Psychiatric Research. 2013;47(5):604-10.

36. Chamberlain SR, Cavanagh J, de Boer P, Mondelli V, Jones DNC, Drevets WC, et al. Treatment-resistant depression and peripheral C-reactive protein. British Journal of Psychiatry. 2019;214(1):11-9.

37. Goodyer IM, Herbert J, Tamplin A, Secher SM, Pearson J. Short-term outcome of major depression .2. Life events, family dysfunction, and friendship difficulties as predictors of persistent disorder. Journal of the American Academy of Child and Adolescent Psychiatry. 1997;36(4):474-80.

38. Schat ACH, Kelloway EK, Desmarais S. The Physical Health Questionnaire (PHQ): Construct validation of a self-report scale of somatic symptoms. Journal of Occupational Health Psychology. 2005;10(4):363-81.

For peer review only - http://bmjopen.bmj.com/site/about/guidelines.xhtml

39. Angold A, Costello EJ, Messer SC, Pickles A, Winder F, Silver D. Development of a short questionnaire for use in epidemiological studies of depression in children and adolescents. International Journal of Methods in Psychiatric Research. 1995;5(4):237-49.

40. Daviss WB, Birmaher B, Melhem NA, Axelson DA, Michaels SM, Brent DA. Criterion validity of the Mood and Feelings Questionnaire for depressive episodes in clinic and non-clinic subjects. Journal of Child Psychology and Psychiatry. 2006;47(9):927-34.

41. Wood A, Kroll L, Moore A, Harrington R. Properties of the mood and feelings questionnaire in adolescent psychiatric outpatients - a research note. Journal of Child Psychology and Psychiatry and Allied Disciplines. 1995;36(2):327-34.

42. Reynolds CR, Richmond BO. What I think and feel: A revised measure of children's manifest anxiety (Reprinted from Journal of Abnormal Child Psychology, vol 6, pg 271-280, 1978). Journal of Abnormal Child Psychology. 1997;25(1):15-20.

43. Tennant R, Hiller L, Fishwick R, Platt S, Joseph S, Weich S, et al. The Warwick-Edinburgh mental well-being scale (WEMWBS): development and UK validation. Health and Quality of Life Outcomes. 2007;5.

44. Goodman R. The strengths and difficulties questionnaire: A research note. Journal of Child Psychology and Psychiatry and Allied Disciplines. 1997;38(5):581-6.

45. van Harmelen AL, Gibson JL, St Clair MC, Owens M, Brodbeck J, Dunn V, et al. Friendships and Family Support Reduce Subsequent Depressive Symptoms in At-Risk Adolescents. Plos One. 2016;11(5).

46. van Harmelen AL, Kievit RA, Ioannidis K, Neufeld S, Jones PB, Bullmore E, et al. Adolescent friendships predict later resilient functioning across psychosocial domains in a healthy community cohort. Psychological Medicine. 2017;47(13):2312-22.

47. Cohen S, Kamarck T, Mermelstein R. A global measure of percieved stress. Journal of Health and Social Behavior. 1983;24(4):385-96.

For peer review only - http://bmjopen.bmj.com/site/about/guidelines.xhtml

48. Russell D, Peplau LA, Cutrona CE. THE REVISED UCLA LONELINESS SCALE - CONCURRENT AND DISCRIMINANT VALIDITY EVIDENCE. Journal of Personality and Social Psychology. 1980;39(3):472-80.

49. Ladouceur CD. COVID-19 Adolescent Symptom & Psychological Experience (CASPE) Questionnaire. 2020.

50. McArdle JJ, Epstein D. Latent growth-curves within developmental structural equation models. Child Development. 1987;58(1):110-33.

51. Muthen BO, Curran PJ. General longitudinal modeling of individual differences in experimental designs: A latent variable framework for analysis and power estimation. Psychological Methods. 1997;2(4):371-402.

52. Moreno-López L, Sallie SN, Ioannidis K, Kaser M, Schueler K, Askelund AD, et al. The RAISE study protocol; a cross-sectional, multi-level, neurobiological study of studying resilience after individual stress exposure. In review.

For peer review only

For peer review only - http://bmjopen.bmj.com/site/about/guidelines.xhtml

SPIRIT

STANDARD PROTOCOL ITEMS: RECOMMENDATIONS FOR INTERVENTIONAL TRIALS

SPIRIT 2013 Checklist: Recommended items to address in a clinical trial protocol and related documents*

Section/item	ItemNo	Description	Addressed on page number
**Administrative information**			
Title	1	Descriptive title identifying the study design, population, interventions, and, if applicable, trial acronym	1
Trial registration	2a	Trial identifier and registry name. If not yet registered, name of intended registry	2
	2b	All items from the World Health Organization Trial Registration Data Set	N/A
Protocol version	3	Date and version identifier	N/A
Funding	4	Sources and types of financial, material, and other support	19
Roles and responsibilities	5a	Names, affiliations, and roles of protocol contributors	18
	5b	Name and contact information for the trial sponsor	19
	5c	Role of study sponsor and funders, if any, in study design; collection, management, analysis, and interpretation of data; writing of the report; and the decision to submit the report for publication, including whether they will have ultimate authority over any of these activities	19
	5d	Composition, roles, and responsibilities of the coordinating centre, steering committee, endpoint adjudication committee, data management team, and other individuals or groups overseeing the trial, if applicable (see Item 21a for data monitoring committee)	18
**Introduction**			
Background and rationale	6a	Description of research question and justification for undertaking the trial, including summary of relevant studies (published and unpublished) examining benefits and harms for each intervention	5-9
	6b	Explanation for choice of comparators	5-9
Objectives	7	Specific objectives or hypotheses	8-9
Trial design	8	Description of trial design including type of trial (eg, parallel group, crossover, factorial, single group), allocation ratio, and framework (eg, superiority, equivalence, noninferiority, exploratory)	4

Methods: Participants, interventions, and outcomes

For peer review only

Study setting	9	Description of study settings (eg, community clinic, academic hospital) and list of countries where data will be collected. Reference to where list of study sites can be obtained	10-11
Eligibility criteria	10	Inclusion and exclusion criteria for participants. If applicable, eligibility criteria for study centres and individuals who will perform the interventions (eg, surgeons, psychotherapists)	10-11
Interventions	11a	Interventions for each group with sufficient detail to allow replication, including how and when they will be administered	N/A
	11b	Criteria for discontinuing or modifying allocated interventions for a given trial participant (eg, drug dose change in response to harms, participant request, or improving/worsening disease)	N/A
	11c	Strategies to improve adherence to intervention protocols, and any procedures for monitoring adherence (eg, drug tablet return, laboratory tests)	N/A
	11d	Relevant concomitant care and interventions that are permitted or prohibited during the trial	N/A
Outcomes	12	Primary, secondary, and other outcomes, including the specific measurement variable (eg, systolic blood pressure), analysis metric (eg, change from baseline, final value, time to event), method of aggregation (eg, median, proportion), and time point for each outcome. Explanation of the clinical relevance of chosen efficacy and harm outcomes is strongly recommended	N/A
Participant timeline	13	Time schedule of enrolment, interventions (including any run-ins and washouts), assessments, and visits for participants. A schematic diagram is highly recommended (see Figure)	9
Sample size	14	Estimated number of participants needed to achieve study objectives and how it was determined, including clinical and statistical assumptions supporting any sample size calculations	10
Recruitment	15	Strategies for achieving adequate participant enrolment to reach target sample size	9
**Methods: Assignment of interventions (for controlled trials)**			
Allocation:			
Sequence generation	16a	Method of generating the allocation sequence (eg, computer-generated random numbers), and list of any factors for stratification. To reduce predictability of a random sequence, details of any planned restriction (eg, blocking) should be provided in a separate document that is unavailable to those who enrol participants or assign interventions	N/A

Allocation concealment mechanism	16b	Mechanism of implementing the allocation sequence (eg, central telephone; sequentially numbered, opaque, sealed envelopes), describing any steps to conceal the sequence until interventions are assigned	N/A
Implementation	16c	Who will generate the allocation sequence, who will enrol participants, and who will assign participants to interventions	N/A
Blinding (masking)	17a	Who will be blinded after assignment to interventions (eg, trial participants, care providers, outcome assessors, data analysts), and how	N/A
	17b	If blinded, circumstances under which unblinding is permissible, and procedure for revealing a participant's allocated intervention during the trial	N/A
**Methods: Data collection, management, and analysis**			
Data collection methods	18a	Plans for assessment and collection of outcome, baseline, and other trial data, including any related processes to promote data quality (eg, duplicate measurements, training of assessors) and a description of study instruments (eg, questionnaires, laboratory tests) along with their reliability and validity, if known. Reference to where data collection forms can be found, if not in the protocol	11-14
	18b	Plans to promote participant retention and complete follow-up, including list of any outcome data to be collected for participants who discontinue or deviate from intervention protocols	9
Data management	19	Plans for data entry, coding, security, and storage, including any related processes to promote data quality (eg, double data entry; range checks for data values). Reference to where details of data management procedures can be found, if not in the protocol	14-15
Statistical methods	20a	Statistical methods for analysing primary and secondary outcomes. Reference to where other details of the statistical analysis plan can be found, if not in the protocol	14-15
	20b	Methods for any additional analyses (eg, subgroup and adjusted analyses)	14-15
	20c	Definition of analysis population relating to protocol non-adherence (eg, as randomised analysis), and any statistical methods to handle missing data (eg, multiple imputation)	14-15
**Methods: Monitoring**			
Data monitoring	21a	Composition of data monitoring committee (DMC); summary of its role and reporting structure; statement of whether it is independent from the sponsor and competing interests; and reference to where further details about its charter can be found, if not in the protocol. Alternatively, an explanation of why a DMC is not needed	N/A

For peer review only - http://bmjopen.bmj.com/site/about/guidelines.xhtml

	21b	Description of any interim analyses and stopping guidelines, including who will have access to these interim results and make the final decision to terminate the trial	N/A
Harms	22	Plans for collecting, assessing, reporting, and managing solicited and spontaneously reported adverse events and other unintended effects of trial interventions or trial conduct	15-16
Auditing	23	Frequency and procedures for auditing trial conduct, if any, and whether the process will be independent from investigators and the sponsor	N/A
**Ethics and dissemination**			
Research ethics approval	24	Plans for seeking research ethics committee/institutional review board (REC/IRB) approval	15-16
Protocol amendments	25	Plans for communicating important protocol modifications (eg, changes to eligibility criteria, outcomes, analyses) to relevant parties (eg, investigators, REC/IRBs, trial participants, trial registries, journals, regulators)	N/A
Consent or assent	26a	Who will obtain informed consent or assent from potential trial participants or authorised surrogates, and how (see Item 32)	9
	26b	Additional consent provisions for collection and use of participant data and biological specimens in ancillary studies, if applicable	N/A
Confidentiality	27	How personal information about potential and enrolled participants will be collected, shared, and maintained in order to protect confidentiality before, during, and after the trial	16
Declaration of interests	28	Financial and other competing interests for principal investigators for the overall trial and each study site	19
Access to data	29	Statement of who will have access to the final trial dataset, and disclosure of contractual agreements that limit such access for investigators	18
Ancillary and post-trial care	30	Provisions, if any, for ancillary and post-trial care, and for compensation to those who suffer harm from trial participation	N/A
Dissemination policy	31a	Plans for investigators and sponsor to communicate trial results to participants, healthcare professionals, the public, and other relevant groups (eg, via publication, reporting in results databases, or other data sharing arrangements), including any publication restrictions	16-17
	31b	Authorship eligibility guidelines and any intended use of professional writers	16-17
	31c	Plans, if any, for granting public access to the full protocol, participant-level dataset, and statistical code	16-17

For peer review only

For peer review only - http://bmjopen.bmj.com/site/about/guidelines.xhtml

Appendices

Informed consent materials	32	Model consent form and other related documentation given to participants and authorised surrogates	N/A
Biological specimens	33	Plans for collection, laboratory evaluation, and storage of biological specimens for genetic or molecular analysis in the current trial and for future use in ancillary studies, if applicable	N/A

*It is strongly recommended that this checklist be read in conjunction with the SPIRIT 2013 Explanation & Elaboration for important clarification on the items. Amendments to the protocol should be tracked and dated. The SPIRIT checklist is copyrighted by the SPIRIT Group under the Creative Commons "Attribution-NonCommercial-NoDerivs 3.0 Unported" license.

For peer review only
